# Long-Term Home Noninvasive Mechanical Ventilation Increases Systemic Inflammatory Response in Chronic Obstructive Pulmonary Disease: A Prospective Observational Study

**DOI:** 10.1155/2014/503145

**Published:** 2014-05-25

**Authors:** Gregorino Paone, Vittoria Conti, Giuseppe Biondi-Zoccai, Elena De Falco, Isotta Chimenti, Mariangela Peruzzi, Corrado Mollica, Gianluca Monaco, Gilda Giannunzio, Giuseppe Brunetti, Giovanni Schmid, V. Marco Ranieri, Giacomo Frati

**Affiliations:** ^1^Department of Cardiovascular, Respiratory, Nephrologic, Anesthesiological, and Geriatric Sciences, Sapienza University of Rome, Viale del Policlinico 155, 00161 Rome, Italy; ^2^Department of Respiratory Diseases, San Camillo-Forlanini Hospital, Circonvallazione Gianicolense 87, 00152 Rome, Italy; ^3^Department of Respiratory Diseases, IRCCS San Raffaele Pisana, Via della Pisana 235, 00163 Rome, Italy; ^4^Department of Medical-Surgical Sciences and Biotechnologies, Sapienza University of Rome, Corso della Repubblica 79, 04100 Latina, Italy; ^5^IRCCS Fondazione Don Carlo Gnocchi-Onlus, Via Maresciallo Caviglia 30, 00194 Rome, Italy; ^6^Department of Anesthesia and Intensive Care Medicine, S. Giovanni Battista Molinette Hospital, University of Turin, Corso Dogliotti 14, 10126 Turin, Italy; ^7^Department of AngioCardioNeurology, IRCCS Neuromed, Pozzilli, Via Atinense 18, Pozzilli, 86077 Isernia, Italy

## Abstract

*Background*. Long-term home noninvasive mechanical ventilation (NIV) is beneficial in COPD but its impact on inflammation is unknown. We assessed the hypothesis that NIV modulates systemic and pulmonary inflammatory biomarkers in stable COPD. *Methods*. Among 610 patients referred for NIV, we shortlisted those undergoing NIV versus oxygen therapy alone, excluding subjects with comorbidities or non-COPD conditions. Sputum and blood samples were collected after 3 months of clinical stability and analyzed for levels of human neutrophil peptides (HNP), interleukin-6 (IL-6), interleukin-10 (IL-10), and tumor necrosis factor-alpha (TNF-alpha). Patients underwent a two-year follow-up. Unadjusted, propensity-matched, and pH-stratified analyses were performed. *Results*. Ninety-three patients were included (48 NIV, 45 oxygen), with analogous baseline features. Sputum analysis showed similar HNP, IL-6, IL-10, and TNF-alpha levels (*P* > 0.5). Conversely, NIV group exhibited higher HNP and IL-6 systemic levels (*P* < 0.001) and lower IL-10 concentrations (*P* < 0.001). Subjects undergoing NIV had a significant reduction of rehospitalizations during follow-up compared to oxygen group (*P* = 0.005). These findings were confirmed after propensity matching and pH stratification. *Conclusions*. These findings challenge prior paradigms based on the assumption that pulmonary inflammation is *per se* detrimental. NIV beneficial impact on lung mechanics may overcome the potential unfavorable effects of an increased inflammatory state.

## 1. Introduction

Noninvasive mechanical ventilation (NIV) reduces erase the need for intubation, length of in-hospital stay, and mortality rate and, therefore, represents the gold standard treatment for patients with chronic obstructive pulmonary disease (COPD) and acute hypercapnic respiratory failure [1–3].

Patients with severe COPD may develop a form of chronic hypercapnic respiratory failure that requires frequent and expensive medical interventions and significantly worsens prognosis and health-related quality of life [4, 5]. Use of NIV in patients with chronic hypercapnic respiratory failure is still under debate [6–10].

Several experimental and clinical studies demonstrated that invasive mechanical ventilation modulates pulmonary and systemic inflammatory responses in healthy [11, 12] or injured lungs [13–18], sustaining the paradigm that overexpression of the inflammatory response is related to poor outcome [19]. However, the impact of these findings in patients on NIV has never been explored. The present study aimed to examine the hypothesis that domiciliary use of NIV may affect pulmonary and systemic inflammatory response in stable COPD patients.

## 2. Material and Methods

The study was conducted in the Respiratory Intermediate Intensive Care Unit (RIICU) of S. Camillo-Forlanini Hospital from March 2007 to January 2010. The Institutional Review Board approved the protocol (no. 584/CE), and written informed consent was obtained from participants.

### 2.1. Patients

Patients with severe and very severe COPD [20], admitted for acute exacerbation and discharged from RIICU with the indication for long-term home NIV, were enrolled.

Inclusion criteria were (1) FEV_1_  < 50% predicted, <20% improvement in FEV_1_ following bronchodilator and a ratio FEV_1_/FVC < 0.70; (2) need for noninvasive mechanical ventilation during an episode of acute respiratory failure; and (3) clinical stability associated with symptoms of nocturnal hypoventilation and PaCO_2_  > 50 mmHg measured immediately after awakening from a night without mechanical ventilation [7–10, 21].

Exclusion criteria were (1) significant comorbidities (e.g., cancer, left ventricular heart failure, and unstable angina) likely to affect survival during follow-up period; (2) psychiatric disorders that could affect the ability to undergo NIV; (3) any other chronic respiratory disease that could interfere with data analysis (e.g., fibrothorax, scoliosis, bronchiectasis, cystic fibrosis, and pulmonary fibrosis); (4) history of obstructive sleep apnoea syndrome (OSAS); (5) body mass index > 40 kg/m^2^; and (5) systemic steroids therapy.

All participants received similar in-hospital management (including an NIV trial before enrollment) and the same home pharmacological treatment (bronchodilators, anticholinergics, and inhaled corticosteroids) to achieve optimal symptoms control as recommended [20].

Among patients meeting the criteria for home NIV, we evaluated 2 subsets of individuals: a study group undergoing home NIV plus long-term oxygen therapy (LTOT) and a control group in treatment with LTOT alone, on the basis of their compliance to NIV treatment (defined as the use of ventilator for ≥5 hrs/night) and/or their willingness to be trained [21].

### 2.2. NIV Protocol

Patients were ventilated using the pressure support ventilation (PSV) module of two ventilators (Neftis; Linde, Munich Germany or Synchrony; Philips Respironics, Andover MA, USA).

Inspiratory positive airway pressure (IPAP) was set as the maximum inspiration pressure value tolerated by patients, able to ensure an exhaled tidal volume of 6 mL/kg (measured body weight). Expiratory positive airway pressure (EPAP) between 2 and 8 cmH_2_O was applied. A back up respiratory rate of 12 breaths/min was set. Oxygen was added to ventilator at a flow able to reach a target SaO_2_≥ 90%. PSV was delivered using either nasal or full face mask based on patient comfort.

### 2.3. Study Evaluations

Four weeks after discharge, a research nurse reached all participants by phone to ascertain their live/dead status and to inquire about the use of NIV (hours per day). After 3 months, surviving patients, free from exacerbations for at least 4 weeks, were asked to return to the hospital for arterial blood gas measurements (ABL 800, Radiometer, Copenhagen) and assessment of pulmonary function tests (PFTs) (Quark PFT Cosmed, Pavona, Italy) [22] and to collect sputum and blood samples. COPD exacerbation was defined following guidelines [23].

Bronchodilator responsiveness to inhaled 400 *μ*g of salbutamol was measured and postbronchodilator values were used [20]. The number of hospital admissions during the previous 2 years was also collected.

Self-assessed smoking cessation was validated by determination of carboxyhemoglobin (COHb) concentration in blood gas analysis and confirmed by interviews with household members [24].

Subsequently, participants entered a 2-year follow-up with regular clinical evaluations carried out every 2 months; hospital admittances number and survival rate were finally recorded.

### 2.4. Blood and Sputum Processing and Analyses

Fasting peripheral blood was collected and samples were stored at −80°C until protein quantification assays. Sputum induction was obtained using 4.5% sodium chloride solution given as two nebulisations each lasting for 7 minutes [25]. Samples were collected and processed within 2 hours. Briefly, sputum was incubated for 15 minutes with four times its weight of freshly prepared 0.1% dithiothreitol (DTT) in Hank's Buffered Salt Solution (HBSS). After incubation the volume of HBSS was doubled and incubated for 5 additional minutes. The suspension was then filtered through a 50 *μ*m nylon gauze to remove mucus and debris and centrifuged at 2000 rpm for 10 minutes. Total cell counts were obtained by using a haemocytometer and cell viability was determined by trypan blue exclusion method. Sputum samples adequacy was evaluated following the literature [26]. Samples were then frozen at −80°C. Concentrations of human neutrophil peptides (HNP), interleukin-6 (IL-6), interleukin-10 (IL-10), and tumor necrosis factor-alpha (TNF-alpha) in sputum and blood samples were quantified by commercial sandwich ELISA following manufacturer instructions (R & D Minneapolis, MN, and HbtCell Sciences, Canton, MA, USA).

### 2.5. Statistical Analysis

Continuous variables are reported as median (1st–3rd quartile) and categorical variables are reported as *n* (%). Continuous variables were compared with Mann-Whitney test for unpaired variables and Wilcoxon test for paired variables, and categorical variables were compared with the chi-squared test (or Fisher's exact test when appropriate). Correlation was appraised with Spearman test. Survival curves were computed with the Kaplan-Meier method and compared with the log-rank test. Propensity score matched-pairs were obtained with a nonparsimonious logistic regression model and used for adjusted analyses. Statistical significance was set at the 2-tailed 0.05 level, and *P* values unadjusted for multiplicity are reported throughout.

## 3. Results

**Figure 1:**
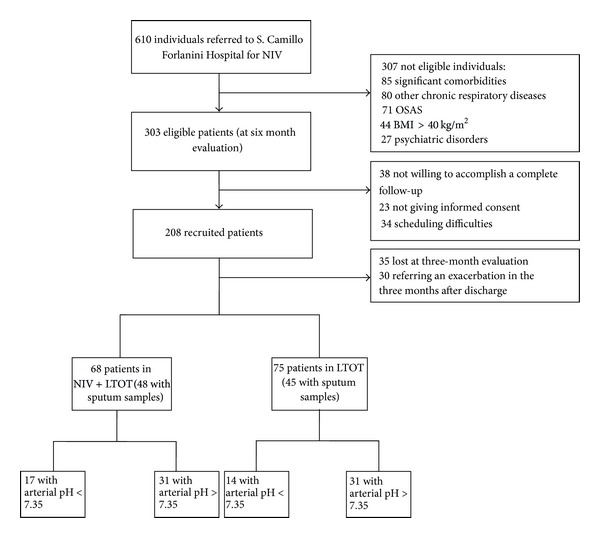
Study flow-chart.

A total of 610 consecutive individuals were referred to our center for an episode of acute respiratory failure; in the population of 459 COPD patients, stage GOLD III and IV, 156 subjects did not satisfy the inclusion criteria because of the presence of significant comorbidities (54.5%), a BMI > 40 kg/m^2^ (28.2%), and psychiatric disorders that could affect ability to undergo NIV (17.3%). Among the 303 eligible individuals, 208 subjects were recruited in the study (Figure 1). Blood and sputum samples were obtained from 48 individuals in NIV + LTOT and 45 patients in LTOT alone.

Patients' baseline demographic data and clinical characteristics are summarized in Table 1. No differences (demographics, smoking history, comorbidities, therapy, pulmonary function, and gas exchange) were observed between study and control group, before and after matching.

Average NIV setting in the study group was IPAP 18.5 ± 2.66 cmH_2_O and EPAP 3.9 ± 1 cmH_2_O. Mean daily use of ventilator was 7.4 ± 1.3 hours.

### 3.1. Inflammatory Biomarkers Measurements

Blood and sputum levels of TNF-alpha, IL-6, IL-10, and HNP observed in the 2 groups are shown in Table 2. Systemic concentrations of HNP and IL-6 were significantly higher, while IL-10 concentrations were lower in patients undergoing home NIV compared to subjects in long-term oxygen therapy (*P* < 0.001); no differences were found in TNF-alpha levels. These findings were confirmed after matching analysis. No significant differences were observed in sputum markers levels between the 2 groups of individuals before and following matching analysis.

Participants were further stratified into 2 subsets according to pH values at initial evaluation (pH < 7.35 or pH > 7.35). The clinical characteristics of the 2 subpopulations are shown in Table 3: no differences in demographics, comorbidities, treatment, lung function, and gas exchange were detected. Systemic higher HNP and IL-6 and lower IL-10 concentrations were observed in subjects with pH < 7.35 undergoing NIV; similar observations were obtained in individuals with pH > 7.35, with the exception of IL-10 (no significant differences). Sputum biomarkers levels were similar between the two subsets of patients (Table 4).

No correlations were found between biomarkers levels and pulmonary function tests (*P* > 0.05); NIV settings analysis pointed out an inverse association between HNP sputum concentrations and EPAP (rho = −0.31, *P* = 0.03).

Sputum and blood biomarkers levels were similar between smokers and nonsmokers (all *P* > 0.05).

No differences were found in the prevalence of individuals with frequent exacerbations (≥2 in the year prior to baseline evaluation) between study and control groups (46% versus 40%, resp., *P* = 0.5). IL-10 sputum levels were significantly decreased in frequent exacerbators as compared to individuals with a low number of exacerbations (5 [3–15] versus 15 [5–23], *P* = 0.02).

### 3.2. Hospitalizations and Survival Rates

Median follow-up period was the same for both groups of participants (24 months). During the follow-up period, hospitalization rates were significantly different between the two groups (1 [0–2] for NIV + LTOT and 2.0 [1–4] for LTOT, *P* = 0.01) with a significant reduction in hospital admissions after enrollment in NIV + LTOT group (2.5 [1–4] versus 1 [0–2], *P* < 0.01) and no differences in LTOT subset (2 [1–3] versus 2 [1–4], *P* = 0.4).

Survival rate was similar between the two groups (27.1% and 22.2%; subjects died in the study and control group, resp., *P* = 0.6), as well as survival time, which appeared to be comparable between the 2 subsets of individuals (22.6 [20.7–24.5] months versus 24.1 [22.5–25.8] months, resp.). In both groups deaths were mostly caused by acute or chronic respiratory failure (34% in NIV + LTOT and 33% in LTOT group), heart failure (22% and 17%, resp.), or pulmonary infection (11% in NIV + LTOT and 17% in LTOT group).

Follow-up results are shown in Table 5.

## 4. Discussion

The present study shows that systemic concentration of inflammatory mediators is higher in patients treated with long-term home noninvasive mechanical ventilation than in patients treated with domiciliary oxygen supplementation only.

While the role of NIV in the management of COPD acute exacerbations is well established [1–3], the impact of long-term home mechanical ventilation is still a matter of debate and its rationale is controversial. Kolodziej et al. concluded that NIV use in patients with severe stable COPD may improve gas exchange, dyspnoea, exercise tolerance, work of breathing, health-related quality of life, and functional status with a significant reduction of the hospitalization rate [7].

Mc Evoy and coworkers showed that NIV was associated with survival improvement while no changes in arterial blood gas analysis, pulmonary function, or hospitalization rates were observed [6]. Recently, a “high intensity” NIV approach was shown to be effective in decreasing PaCO_2_, improving lung function and global inspiratory muscle strength [27, 28].

The ability of conventional invasive mechanical ventilation to initiate or worsen pulmonary and systemic inflammatory response has been demonstrated in experimental and clinical settings. These data led to the hypothesis that mechanical ventilation (MV) may contribute to worsen or cause lung injury [13, 17, 18] and may be related to the development of multiple organ failure. Although current research concerning ventilator induced lung injury (VILI) is largely based on positive pressure ventilation delivered via endotracheal tube, these principles may be equally relevant to noninvasive pressure ventilation [29].

While very few studies have evaluated the role of NIV in pulmonary and systemic inflammation in animal models and humans [30, 31], to the best of our knowledge, this is the first report aimed at mutually analyzing local and systemic inflammatory responses in patients undergoing long-term NIV for stable COPD.

We found a significant increase in IL-6 and HNP systemic concentrations together with a noteworthy lower amount of IL-10 in patients undergoing long-term NIV.

It is important to underline that the systemic levels of proinflammatory molecules we found in our report, although increased in NIV population, were by far lower than those reported in studies involving patients with invasive ventilation-associated lung injury [12, 18] and in the range of concentrations observed in individuals with stable COPD [32].

Cytokines are low-molecular weight proteins that may initiate and orchestrate inflammatory response to different stimuli. They are produced by airway epithelial cells, alveolar macrophages, neutrophils, and lymphocytes. Concentration of IL-6 has been shown to be increased during positive mechanical ventilation-associated lung injury and its role in VILI is well established [18, 33, 34].

HNPs represent more than 30% of azurophilic granules content and stimulate alveolar macrophages to release IL-8, leukotriene B4 (LTB4), and TNF-alpha [35, 36] which may determine a vicious circle contributing to perpetuate inflammation.

The balance between proinflammatory (IL-6) and anti-inflammatory (IL-10) cytokines is crucial in regulating the immune response, contributing to the dampening of the otherwise massive inflammatory response in the lower respiratory tract [37].

In order to obtain a more homogeneous sample and to avoid the profound impact that COPD exacerbations could have had on lung and systemic inflammatory responses, we excluded from the evaluation subjects with any symptom or sign of exacerbation [38]. In addition, because of the pivotal role of pH in COPD patients with respiratory failure, we further stratified participants using a pH cut-off value of 7.35 [6, 8]. The differences between systemic markers concentrations were maintained, confirming the overall evaluation. The clinical follow-up evaluation after 2 years, although not showing differences in survival between the 2 groups, pointed out a significant decrease in the rate of hospital admissions in the study group during the follow-up period, in line with other reports [7, 8].

Our study has several limitations. First, it is an observational cohort study. Accordingly, the lack of randomization remains a key flaw of this work, given the inherent risks of selection, performance, attrition, and adjudication bias. Nonetheless, carefully designed observational studies may reduce the risk of imprecise and inaccurate estimates [39]. To minimize the risk of overestimating or underestimating biologic and clinical effects, we relied for both groups on established indications criteria for the use of NIV in stable COPD [6, 8, 10, 21]. In addition, the choice of medical treatment and ventilatory settings did not significantly change over the period of interest in our institution, providing a common management ground for our comparisons. Moreover, in order to reduce potential confounding factors, propensity score matching and pH-based stratified analyses were performed to compensate for nonrandom assignment to treatments. Second, due to the high number of exacerbations observed during the study period, we did not achieve a time course collection of biomarkers from an adequate number of patients. Therefore, we could not evaluate over time the effects of mechanical ventilation on the modifications of markers concentrations.

Our study is the first analyzing biomarkers levels in COPD patients undergoing long-term home NIV. We reckon that our major finding is that patients with NIV have a significant increase in systemic inflammation as compared to a control group undergoing LTOT alone. Remarkably, follow-up analysis showed a significant lower hospitalization in the study group as compared to control group. Therefore this data seems to suggest that, at least in patients with stable COPD, the activation of proinflammatory mediators related to mechanical ventilation is not linked to an unfavorable clinical outcome. A similar role of a proinflammatory response necessary for adaptive cardiac remodeling observed in the cardiovascular system may explain our preliminary findings [40–42].

In conclusion, this preliminary study provides original information regarding the relationship between NIV and inflammatory response in patients with chronic hypercapnic respiratory failure. Our data might challenge the view that activation of a proinflammatory signal is* per se* related to a worse clinical outcome. In this context we cannot rule out that the beneficial impact of NIV on respiratory mechanics (reduction of hyperinflation, work of breathing, and respiratory muscles overload) may overcome the potential unfavorable effects of an increased inflammatory state.

Further studies are required to confirm these preliminary observations.

## Figures and Tables

**Table 1:** Baseline features*.

	Long-term oxygen therapy	Noninvasive ventilation	*P* value
Before matching	*N* = 45	*N* = 48	

Age (years)	72 (66–78)	69 (64–74)	0.091
Male gender	23 (51.1%)	21 (43.8%)	0.477
Diabetes mellitus	7 (15.6%)	7 (14.6%)	1.0
Arterial hypertension	36 (80.0%)	46 (95.8%)	0.018
Smoking status	4 (8.9%)	3 (6.2%)	0.709
Cor pulmonale	11 (24.4%)	11 (22.9%)	1.0
Long-acting muscarinic agent only	2 (4.4%)	1 (2.1%)	0.609
Long-acting beta2 agonist only	0	1 (2.1%)	1.0
Inhalatory corticosteroid only	0	0	1.0
Long-acting beta2 agonist plus inhalatory corticosteroid	6 (13.3%)	4 (8.3%)	0.515
Long-acting muscarinic agent, plus long-acting beta2 agonist and inhalatory corticosteroid	37 (82.2%)	41 (85.4%)	0.676
Oxygen therapy (L/min)	2.0 (2.0–3.0)	2.5 (2.0–3.0)	0.059
FEV_1_ (% predicted)	30.0 (23.5–34.5)	27.5 (23.0–34.8)	0.467
FVC (% predicted)	52.0 (47.0–63.0)	50.0 (42.8–57.0)	0.120
FEV_1_/FVC (%)	54.0 (48.0–61.5)	57.0 (50.0–63.5)	0.385
pH	7.36 (7.34–7.38)	7.35 (7.34–7.37)	0.125
PO_2_ (mm Hg)	72.2 (62.5–84.4)	72.4 (66.6–80.9)	0.756
PCO_2_ (mm Hg)	55.6 (48.7–61.9)	57.8 (52.9–67.3)	0.086
Oxygen saturation (%)	93.6 (92.8–95.4)	93.9 (92.6–96.1)	0.732
White blood cells (cell number × 10^3^/mL)	8.00 (6.85–9.59)	7.73 (6.25–8.48)	0.227
Polymorphonuclear neutrophils (%)	71.3 (66.0–75.4)	66.4 (60.4–73.1)	0.020
Procalcitonin (ng/mL)	<0.05	<0.05	1.0

After matching	*N* = 30	*N* = 30	

Age (years)	71 (66–77)	70 (64–73)	0.390
Male gender	15 (50.0%)	16 (53.3%)	0.796
Diabetes mellitus	4 (13.3%)	3 (10.0%)	1.0
Arterial hypertension	29 (96.7%)	29 (96.7%)	1.0
Smoking status	2 (6.7%)	1 (3.3%)	1.0
Cor pulmonale	8 (26.7%)	9 (30.0%)	0.774
Long-acting muscarinic agent only	1 (3.3%)	1 (3.3%)	1.0
Long-acting beta2 agonist only	0	0	1.0
Inhalatory corticosteroid only	0	0	1.0
Long-acting beta2 agonist plus inhalatory corticosteroid	4 (13.3%)	2 (6.7%)	0.671
Long-acting muscarinic agent, plus long-acting beta2 agonist and inhalatory corticosteroid	25 (83.3%)	27 (90.0%)	0.706
Oxygen therapy (L/min)	3.0 (2.0–4.3)	2.3 (2.0–3.0)	0.837
FEV_1_ (% predicted)	27.5 (22.8–32.5)	28.0 (23.8–35.0)	0.695
FVC (% predicted)	51.5 (44.8–61.5)	50.0 (45.0–57.0)	0.351
FEV_1_/FVC (%)	54.0 (47.0–61.3)	59.0 (50.0–64.3)	0.228
pH	7.36 (7.33–7.37)	7.35 (7.34–7.36)	0.466
PO_2_ (mm Hg)	68.5 (62.4–80.1)	73.3 (68.0–81.0)	0.176
PCO_2_ (mm Hg)	55.7 (48.7–61.4)	57.8 (54.2–64.1)	0.139
Oxygen saturation (%)	93.4 (92.8–94.8)	94.1 (93.0–96.0)	0.228
White blood cells (cell number × 10^3^/mL)	8.03 (6.68–9.83)	7.72 (5.73–8.38)	0.154
Polymorphonuclear neutrophils (%)	72.2 (67.2–75.5)	66.0 (60.2–70.7)	0.011
Procalcitonin (ng/mL)	<0.05	<0.05	1.0

*reported as median (1st–3rd quartile) or *n* (%) and compared with Mann-Whitney *U*-test, chi-squared test, or Fisher's exact test.

**Table 2:** Blood and sputum biomarkers concentrations*.

	Long-term oxygen therapy	Noninvasive ventilation	*P* value
Before matching	*N* = 45	*N* = 48	

Sputum			
Human neutrophil peptides (*µ*g/mL)	34.5 (33.0–35.3)	34.0 (32.3–36.0)	0.787
Interleukin-6 (pg/mL)	40.0 (19.0–51.5)	41.9 (18.0–68.5)	0.275
Interleukin-10 (pg/mL)	14.0 (6.0–24.0)	5.0 (4.0–20.0)	0.092
Tumor necrosis factor-alpha (pg/mL)	32.0 (22.0–110.0)	49.0 (30.0–99.0)	0.412
Blood			
Human neutrophil peptides (*µ*g/mL)	3.3 (1.1–9.8)	10.8 (7.9–11.7)	<0.001
Interleukin-6 (pg/mL)	3.7 (2.9–6.0)	8.2 (6.1–10.9)	<0.001
Interleukin-10 (pg/mL)	7.0 (5.4–8.0)	3.2 (0.8–6.7)	<0.001
Tumor necrosis factor-alpha (pg/mL)	7.0 (3.0–9.0)	8.0 (5.0–10.0)	0.122
Sputum/blood ratio			
Human neutrophil peptides	10.5 (3.7–35.1)	3.3 (2.9–5.2)	<0.001
Interleukin-6	7.0 (4.6–14.4)	3.9 (2.0–8.2)	0.010
Interleukin-10	2.6 (1.0–3.8)	3.5 (0.9–6.6)	<0.001
Tumor necrosis factor-alpha	7.8 (3.7–14.0)	8.0 (3.8–15.0)	0.803

After matching	*N* = 30	*N* = 30	

Sputum			
Human neutrophil peptides (*µ*g/mL)	34.0 (33.0–35.6)	33.6 (31.5–36.0)	0.563
Interleukin-6 (pg/mL)	42.5 (28.3–52.3)	40.9 (16.0–65.5)	0.734
Interleukin-10 (pg/mL)	14.0 (3.8–24.3)	5.5 (3.0–23.5)	0.347
Tumor necrosis factor-alpha (pg/mL)	29.0 (20.0–72.5)	47.0 (27.0–105.0)	0.068
Blood			
Human neutrophil peptides (*µ*g/mL)	3.2 (0.9–10.5)	10.9 (7.2–11.7)	0.003
Interleukin-6 (pg/mL)	3.5 (2.9–6.1)	8.7 (6.3–18.3)	<0.001
Interleukin-10 (pg/mL)	6.5 (5.0–8.0)	1.0 (0.7–6.0)	<0.001
Tumor necrosis factor-alpha (pg/mL)	6.5 (4.5–8.3)	8.0 (4.6–11.8)	0.216
Sputum/blood ratio			
Human neutrophil peptides	11.0 (3.5–41.0)	3.2 (2.9–5.7)	0.002
Interleukin-6	8.5 (5.0–16.1)	3.9 (1.5–8.4)	0.004
Interleukin-10	2.6 (1.1–4.0)	4.2 (1.4–8.8)	0.042
Tumor necrosis factor-alpha	4.9 (2.5–13.4)	8.5 (3.6–15.2)	0.344

*reported as median (1st–3rd quartile) or *n* (%) and compared with Mann-Whitney *U* test, chi-squared test, or Fisher exact test.

**Table 3:** Baseline features according to pH*.

	Long-term oxygen therapy	Noninvasive ventilation	*P* value
pH < 7.35	*N* = 14	*N* = 17	

Age (years)	69 (64–78)	67 (62–72)	0.426
Male gender	6 (42.9%)	10 (58.8%)	0.376
Diabetes mellitus	3 (21.4%)	3 (17.6%)	1.0
Arterial hypertension	12 (85.7%)	17 (100%)	0.196
Smoking status	1 (7.1%)	1 (5.9%)	1.0
Cor pulmonale	2 (14.3%)	3 (17.6%)	1.0
Long-acting muscarinic agent only	2 (14.3%)	1 (5.9%)	0.576
Long-acting beta2 agonist only	0	0	1.0
Inhalatory corticosteroid only	0	0	1.0
Long-acting beta2 agonist plus inhalatory corticosteroid	0	0	1.0
Long-acting muscarinic agent plus long-acting beta2 agonist and inhalatory corticosteroid	12 (85.7%)	16 (94.1%)	0.576
Oxygen therapy	2.3 (2.0–3.3)	2.0 (2.0–3.0)	0.672
FEV_1_ (% predicted)	30.5 (25.0–32.5)	24.0 (20.0–31.0)	0.209
FVC (% predicted)	56.5 (43.8–61.5)	48.0 (40.5–52.0)	0.108
FEV_1_/FVC (%)	51.0 (46.3–61.3)	55.0 (45.5–62.5)	0.633
pH	7.32 (7.31–7.33)	7.33 (7.31–7.34)	0.382
PO_2_ (mm Hg)	73.6 (66.3–83.9)	72.0 (66.0–79.0)	0.648
PCO_2_ (mm Hg)	64.2 (55.1–67.5)	67.9 (59.2–71.0)	0.266
Oxygen saturation (%)	94.4 (93.2–95.6)	93.3 (91.5–95.4)	0.275
White blood cells (cell number × 10^3^/mL)	7.67 (6.74–9.47)	7.60 (5.45–8.46)	0.147
Polymorphonuclear neutrophils (%)	69.9 (67.2–73.6)	66.4 (61.6–72.3)	0.137
Procalcitonin (ng/mL)	<0.05	<0.05	1.0

pH ≥ 7.35	*N* = 31	*N* = 31	

Age (years)	72 (66–76)	70 (65–74)	0.139
Male gender	17 (54.8%)	11 (35.5%)	0.126
Diabetes mellitus	4 (12.9%)	4 (12.9%)	1.0
Arterial hypertension	24 (77.4%)	29 (93.5%)	0.147
Smoking status	3 (9.7%)	2 (6.5%)	1.0
Cor pulmonale	9 (29.0%)	8 (25.8%)	0.776
Long-acting muscarinic agent only	0	0	1.0
Long-acting beta2 agonist only	0	1 (3.2%)	1.0
Inhalatory corticosteroid only	0	0	1.0
Long-acting beta2 agonist plus inhalatory corticosteroid	6 (19.4%)	4 (12.9%)	0.490
Long-acting muscarinic agent plus long-acting beta2 agonist and inhalatory corticosteroid	25 (80.6%)	25 (80.6%)	1.0
Oxygen therapy	2.0 (2.0–2.5)	3.0 (2.0–4.0)	0.015
FEV_1_ (% predicted)	30.0 (23.0–37.0)	31.0 (26.0–36.0)	0.893
FVC (% predicted)	51.0 (48.0–64.0)	52.0 (46.0–60.0)	0.490
FEV_1_/FVC (%)	57.0 (49.0–62.0)	59.0 (51.0–65.0)	0.434
pH	7.37 (7.36–7.39)	7.36 (7.35–7.38)	0.013
PO_2_ (mm Hg)	71.8 (62.4–85.2)	74.0 (67.0–83.9)	0.593
PCO_2_ (mm Hg)	53.8 (46.8–57.5)	56.0 (50.6–60.3)	0.141
Oxygen saturation (%)	93.3 (92.6–95.5)	94.1 (93.0–96.7)	0.239
White blood cells (cell number × 10^3^/mL)	8.04 (6.84–9.68)	7.80 (6.71–8.87)	0.573
Polymorphonuclear neutrophils (%)	72.1 (64.9–75.6)	65.6 (60.2–73.9)	0.077
Procalcitonin (ng/mL)	<0.05	<0.05	1.0

*reported as median (1st–3rd quartile) or *n* (%) and compared with Mann-Whitney *U*-test, chi-squared test, or Fisher's exact test.

**Table 4:** Blood and sputum biomarkers concentrations according to pH*.

	Long-term oxygen therapy	Noninvasive ventilation	*P* value
pH < 7.35	*N* = 14	*N* = 17	

Sputum			
Human neutrophil peptides (*µ*g/mL)	33.2 (30.8–35.3)	33.0 (30.0–36.0)	0.952
Interleukin-6 (pg/mL)	40.0 (19.8–56.0)	50.0 (23.0–75.5)	0.311
Interleukin-10 (pg/mL)	11.0 (5.3–18.0)	5.0 (4.0–24.0)	0.499
Tumor necrosis factor-alpha (pg/mL)	56.0 (26.5–113.8)	45.0 (22.0–115.0)	0.858
Blood			
Human neutrophil peptides (*µ*g/mL)	2.3 (1.1–9.6)	11.0 (5.4–11.5)	0.040
Interleukin-6 (pg/mL)	4.7 (2.9–6.9)	7.0 (4.0–18.4)	0.049
Interleukin-10 (pg/mL)	7.0 (5.9–8.5)	1.0 (0.7–6.0)	0.001
Tumor necrosis factor-alpha (pg/mL)	6.5 (4.8–8.3)	8.0 (3.4–10.0)	0.617
Sputum/blood ratio			
HNP	16.8 (3.6–39.3)	3.1 (2.8–10.6)	0.032
Interleukin-6	7.9 (5.0–13.6)	7.5 (2.7–11.9)	0.475
Interleukin-10	1.7 (0.8–2.9)	4.1 (0.9–9.0)	0.068
Tumor necrosis factor-alpha	9.5 (3.6–16.0)	8.5 (2.9–17.5)	0.953

pH ≥ 7.35	*N* = 31	*N* = 31	

Sputum			
Human neutrophil peptides (*µ*g/mL)	34.5 (33.0–35.4)	34.0 (33.0–37.2)	0.677
Interleukin-6 (pg/mL)	40.0 (16.0–50.0)	35.0 (16.0–58.0)	0.592
Interleukin-10 (pg/mL)	14.0 (6.0–25.0)	5.0 (4.0–18.0)	0.113
Tumor necrosis factor-alpha (pg/mL)	30.0 (20.0–110.0)	50.0 (31.0–96.0)	0.207
Blood			
Human neutrophil peptides (*µ*g/mL)	3.5 (1.0–10.5)	9.4 (7.8–11.8)	0.002
Interleukin-6 (pg/mL)	3.5 (2.9–6.0)	8.4 (6.3–10.6)	<0.001
Interleukin-10 (pg/mL)	7.0 (1.0–8.0)	3.6 (0.9–7.0)	0.097
Tumor necrosis factor-alpha (pg/mL)	7.0 (2.0–10.0)	8.0 (5.4–14.8)	0.095
Sputum/blood ratio			
HNP	9.4 (3.5–35.1)	3.7 (2.9–5.4)	0.002
Interleukin-6	6.7 (3.8–15.0)	3.3 (1.8–7.5)	0.010
Interleukin-10	3.0 (1.1–4.4)	3.3 (0.8–5.0)	0.751
Tumor necrosis factor-alpha	7.8 (3.7–14.0)	6.1 (3.9–12.5)	0.683

*reported as median (1st–3rd quartile) or *n* (%) and compared with Mann-Whitney *U*-test, chi-squared test, or Fisher's exact test.

**Table 5:** Clinical results*.

	Long-term oxygen therapy	Noninvasive ventilation	*P* value
*Overall population *			
Before matching	*N* = 45	*N* = 48	
Follow-up (months)	24.0 (21.0–25.5)	24.0 (24.0–24.0)	0.190
Prior hospitalizations	2.0 (1.0–3.0)	2.5 (1.0–4.0)	0.498
Subsequent hospitalizations	2.0 (1.0–4.0)	1.0 (0–2.0)	0.005
All hospitalizations	4.0 (2.0–7.0)	4.0 (2.0–6.8)	0.536
Death	10 (22.2%)	13 (27.1%)	0.587
After matching	*N* = 30	*N* = 30	
Follow-up (months)	24.0 (22.5–25.0)	24.0 (22.5–24.0)	0.198
Prior hospitalizations	3.0 (2.0–3.0)	2.0 (1.0–3.3)	0.564
Subsequent hospitalizations	2.5 (1.0–4.0)	1.0 (0–1.3)	<0.001
All hospitalizations	5.0 (3.0–7.0)	3.0 (1.8–5.0)	0.021
Death	7 (23.3%)	9 (30.0%)	0.976
*Excluding cross-overs *			
Before matching	*N* = 38	*N* = 48	
Follow-up (months)	24.0 (16.5–25.0)	24.0 (24.0-24.0)	0.325
Prior hospitalizations	2.0 (1.0–3.0)	2.5 (1.0–4.0)	0.282
Subsequent hospitalizations	2.0 (1.0–3.0)	1.0 (0–2.0)	0.024
All hospitalizations	4.0 (2.0–5.3)	4.0 (2.0–6.8)	0.885
Death	9 (23.7%)	13 (27.1%)	0.720
After matching	*N* = 26	*N* = 30	
Follow-up (months)	24.5 (22.5–25.0)	24.0 (22.5–24.0)	0.168
Prior hospitalizations	2.0 (2.0–3.0)	2.0 (1.0–3.3)	0.980
Subsequent hospitalizations	2.0 (1.0–3.0)	1.0 (0–1.3)	0.001
All hospitalizations	4.5 (3.0–6.0)	3.0 (1.8–5.0)	0.082
Death	6 (23.1%)	9 (30.0%)	0.560

*reported as median (1st–3rd quartile) or *n* (%) and compared with Mann-Whitney *U*-test, chi-squared test, or Fisher's exact test.

## Abbreviations

NIV: Noninvasive mechanical ventilation
COPD: Chronic obstructive pulmonary disease
LTOT: Long-term oxygen therapy
OSAS: Obstructive sleep apnea syndrome
HNPs: Human neutrophil peptides
IL-10: Interleukin-10
IL-6: Interleukin-6
TNF-alpha: Tumor necrosis factor-alpha.
